# A novel textile-based UWB patch antenna for breast cancer imaging

**DOI:** 10.1007/s13246-024-01409-w

**Published:** 2024-03-26

**Authors:** Fawzia Abdien ali abdulla, Aşkin Demirkol

**Affiliations:** Electrical and Electronics Engineering, Sakarya University, Sakarya, 54100 Turkey

**Keywords:** Breast cancer detection, Microstrip patch antenna, UWB microwave imaging, Return loss, Textile-based antenna

## Abstract

Breast cancer is the second leading cause of death for women worldwide, and detecting cancer at an early stage increases the survival rate by 97%. In this study, a novel textile-based ultrawideband (UWB) microstrip patch antenna was designed and modeled to work in the 2–11.6 GHz frequency range and a simulation was used to test its performance in early breast cancer detection. The antenna was designed with an overall size of 31*31 mm ^2^ using a denim substrate and 100% metal polyamide-based fabric with copper, silver, and nickel to provide comfort for the wearer. The designed antenna was tested in four numerical breast models. The models ranged from simple tumor-free to complex models with small tumors. The size, structure, and position of the tumor were modified to test the suggested ability of the antenna to detect cancers with different shapes, sizes, and positions. The specific absorption rate (SAR), return loss (S11), and voltage standing wave ratio (VSWR) were calculated for each model to measure the antenna performance. The simulation results showed that SAR values were between 1.6 and 2 W/g (10 g SAR) and were within the allowed range for medical applications. Additionally, the VSWR remained in an acceptable range from 1.15 to 2. Depending on the size and location of the tumor, the antenna return losses of the four models ranged from $-$36 to $-$18.5 dB. The effect of bending was tested to determine the flexibility. The antenna proved to be highly effective and capable of detecting small tumors with diameters of up to 2 mm.

## Introduction

Breast cancer is a condition in which breast cells start growing and dividing uncontrollably and invade surrounding healthy cells or metastasize into several other body parts [1]. Breast cancer was identified as the second leading cause of death for women worldwide, and there were approximately 685000 deaths in 2020 [2]. According to the World Health Organization, there were more than 2.26 million new cases of breast cancer identified worldwide in 2020; it is the most commonly diagnosed cancer, representing 11.7 of the overall 19.3 million new cancer cases [3]. The American Cancer Society estimates that there will be approximately 297,790 new cases of breast cancer diagnosed in women, and approximately 43,700 will die in 2023 in the United States only [4]. Researchers have emphasized that accurate and highly efficient approaches are needed for breast cancer detection since detecting cancer at an early stage raises the survival rate by up to 97% [5].

Among the various diagnosis platforms, imaging modalities represent the most important parts of cancer diagnosis and treatment operation since they offer a wide range of invisible information and interior body pictures [6]. Breast imaging systems, in general, refer to the commonly used diagnostic methods for detecting breast tumors, such as ultrasonic (U.S.) imaging, mammography, positron emission tomography (PET), and magnetic resonance imaging (MRI) [7, 8]. The drawbacks and limitations of the existing breast imaging approaches inspired researchers to create and develop novel microwave-based techniques [9]. The nonionizing radiation and noninvasive characteristics of microwaves make them a capable option in the field of breast cancer detection [10, 11]. The most commonly utilized methods that expose the body to microwaves and analyze the transmitted and received signals are microwave tomography and UWB radar imaging[12, 13].

Active microwave imaging is based on electromagnetic scattering due to the dielectric contrast between the different objects under investigation [14]. In the situation of breast imaging, the dielectric contrast between different tissue types has been used in active microwave imaging to generate a 2D or 3D image of the breast[15]. Over the last few years, several active microwave breast imaging systems have been developed [16, 17]. Unlike the image reconstruction goal of microwave tomography, UWB’s radar-based imaging system addresses a specific computational challenge, focusing on determining the location of large scattering obstacles such as malignant tumors [18].

The importance of antennas in UWB imaging cannot be overstated, as they are the interface between the electromagnetic waves and the objects being imaged. Some transducers (microwave antennas) that operate in the medical microwave frequency range of 300 MHz to 20 GHz are used to illuminate the breast [19, 20]. These short pulses contain valuable information about the object under investigation. When a tumor is present, it creates backscattered signals at microwave frequencies, which depend on the contrast between healthy and malignant tissues [21]. The system’s resolution capabilities are influenced by UWB antenna properties, enabling the differentiation of small features or anomalies within the object [22].

In breast imaging, it’s essential that the antennas are comfortable and flexible [23]. Patient comfort and compliance are critical factors, and antennas that can conform to the shape of the breast while maintaining performance are crucial for practical and effective imaging. Also, the sensitivity of tumor detection depends on the interspace between the antenna and the breast, and decreasing this distance leads to an increase in detection sensitivity [19]. On the other hand, recent global health crises, such as the COVID-19 pandemic that emerged in 2019, have underscored the importance of seeking alternatives to conventional breast imaging devices [24]. The anticipated progress in the medical device industry is expected to pave the way for out-of-hospital screening, thereby addressing diagnostic delays that may arise from health crises [25]. There has been growing interest in developing flexible and textile-based wearable microwave antennas. Wearable prototypes seem to be more affordable and have considerably smaller overall sizes than table-based devices. Textile substrates are increasingly preferred as the primary materials for wearable biomedical antennas due to their flexibility and comfort for the wearer [26].

Mahmood et al. [27] proposed a fully grounded UWB textile antenna operating in the 7-28 GHz frequency band for early breast cancer detection. They used denim as the substrate and a shield conductive textile for patch and ground with a total size of 60*50*0.7 mm^3^. Bahrami et al [28] introduced a small, flexible monopole antenna for breast imaging that operated within the 2–5 GHz frequency band. Although their antenna was small at 20*20 mm^2^, the narrow band affected the image resolution. Srinivasan et al. [29] presented a novel antenna that utilized a jean substrate and copper conductive material to detect breast cancer, and it operated within the 2.4 GHz frequency band. Other researchers have worked on designing textile-based wearable antennas for on-body applications [30–35].

Wearable antennas have the potential to revolutionize breast cancer screening by providing real-time, portable, and patient-friendly solutions. However, the integration of UWB antennas into wearable textiles presents several technical challenges, including the design of compact and efficient antennas that can maintain their performance when integrated into fabrics, ensuring flexibility and durability, addressing biocompatibility concerns, and ensuring radiation safety [36].

This article addresses these challenges by presenting a novel textile-based UWB patch antenna designed specifically for breast cancer imaging applications. The development of such antennas represents a significant advancement in the field of medical imaging and has the potential to transform the way breast cancer is diagnosed and monitored.

The primary objective of the research is to design a UWB patch antenna using textile materials for both the substrate and conductor. In contrast to conventional studies that typically employ fabric as the substrate and metal for the conductor, this study addresses biocompatibility concerns by utilizing a full textile-based antenna. The main objectives of this antenna design are to achieve compactness and simplicity while maintaining operation within the frequency range of 2–11.6 GHz. Achieving this wide frequency coverage is particularly challenging when using fabric materials in small antennas. This extensive frequency range plays a crucial role in facilitating high penetration and resolution in breast imaging. These properties enable the antenna to detect tumors of various shapes and sizes in different areas of the breast. Unlike many existing models that simplify tumor shapes by assuming they are spherical, this research employs more realistic models and acknowledges the diversity of tumor shapes and sizes encountered in clinical scenarios. To assess the performance of each antenna model, various critical parameters, including specific absorption rate (SAR) for radiation safety, return losses, voltage standing wave ratio (VSWR) to ensure antenna efficiency, and the impact of bending to determine flexibility, were examined.

## Methodology

### Antenna design

**Fig. 1:**
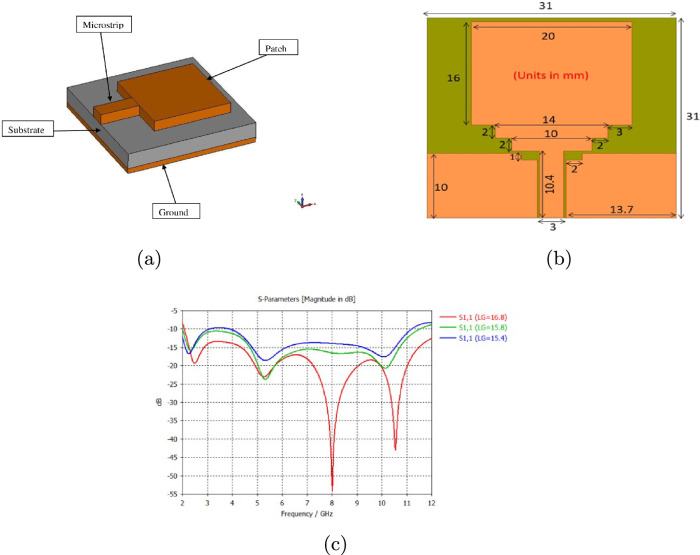
**a** Basic rectangular patch antenna, **b** proposed antenna after the optimization process **c** Impact of the partial ground plane in the return loss

Antenna selection is very important in microwave imaging, and the antenna must be able to transmit signals as accurately and efficiently as possible. In previous research, wearable UWB antennas have shown limitations in resolution, low bandwidth, high SAR values, and larger dimensions. In order to efficiently detect tumors, a wearable antenna designed for breast tumor detection should have a wide bandwidth, low SAR, compact design, and high degree of adaptability. Due to their low-profile conformal designs, low costs, simple manufacturing processes, and adaptabilities in terms of implementation, microstrip patch antennas have been utilized. The patch antenna consists of a conductive ground layer, a dielectric layer above it, and a conducting patch over the substrate, as shown in Fig. 1a. Patch antennas are typically designed to operate at a specific frequency or within a narrow frequency band. However, there are several methods to increase the bandwidth of patch antennas, including increasing the thickness of the substrate, cutting slots, cutting notches, and using a partial ground plane. In this work, bandwidth has been increased by cutting notches, in addition to using a partial ground plan. The proposed rectangular microstrip patch antenna was conceived for a UWB breast cancer imaging application, and it operated in the frequency of range 2–11.6 GHz. The antenna was developed with a jeans substrate with a dielectric $\in _r$ equal to 1.7 and a height (h) of 0.7 mm. A 100% polyamide-based fabric metalized with copper, silver, and nickel was used as the conductive material for the patch and ground plane with a thickness of 0.11 mm. The dimensions of a patch antenna play a fundamental role in determining its characteristics and performance. The length and width of the patch determine the resonant frequency of the antenna. Generally, a longer patch corresponds to a lower resonant frequency, while a wider patch results in a higher resonant frequency. Adjusting L and W allows the antenna to operate at a specific frequency or within a desired frequency band. Figure 1c shows how the return loss of an antenna changes as the length of the partial ground plane is varied. The most commonly used equations for calculating L and W are based on the fundamental resonant mode of the patch, which is the half-wavelength mode. Equations 1 through 6 indicated the primary antenna dimensions [37]. Once the desired resonant frequency and dielectric constant are known, Equation (1) is used to determine the patch width (W).

1 $$\begin{aligned} W=\frac{C}{2f_r}\sqrt{\frac{2}{\in _r+1}} \end{aligned}$$

where C is the velocity of light, $3*10^8m/s$, $\in _r$ is the substrate dielectric constant, and *fr* the antenna resonant frequency.

After determining the width, there are additional parameters that need to be considered to calculate the length of the patch accurately. These parameters include the effective dielectric constant, the effective length, and the antenna length extension. These are necessary for more precise design and impedance matching. The effective dielectric constant ($\in _eff$) was calculated with Eq. (2).

2 $$\begin{aligned} \in _{eff}=\frac{\in _r+1}{2}+\frac{\in _r-1}{2}[\frac{1}{\sqrt{(1+\frac{12h}{W}}} \end{aligned}$$

The effective length (Leff) was calculated as

3 $$\begin{aligned} L_{eff}=\frac{C}{2f_r\sqrt{\in _eff}} \end{aligned}$$

The antenna length extension ($\Delta L$) was calculated as

4 $$\begin{aligned} \Delta L=h*0.412*\left[ \frac{(\in _{eff}+0.3)\left[ \frac{W}{h}+0.264\right] }{(\in _{eff}-0.258) \left[ \frac{W}{h}+0.8\right] }\right] \end{aligned}$$

To determine the antenna length (L),

5 $$\begin{aligned} L=L_{eff}-2\Delta L \end{aligned}$$

Antennas are designed to be used in free space; therefore, antenna theory does not work correctly with human tissues, which requires optimizing the antenna design to make it compatible with human tissues. The return loss changed when the antenna was inserted into a breast (high permittivity medium). Miniaturization techniques are used for minimum return loss, especially with the antenna ground, and slot length. The antenna optimization process was performed to achieve the desired bandwidth. The trust region framework (TRF) algorithm was used with the electromagnetic (EM) Computer Simulation Technology (CST) software to optimize the ground plane length to achieve the desired bandwidth range of 2–11.6 GHz. The designed and optimized antenna dimensions are shown in Fig. 1b and Table 1, respectively. The microstrip line feeding method was used to feed the antenna.

**Table 1:** Optimized antenna dimensions

Parameters 1	Dimensions (mm)
Patch width	20
Patch length	20
Substrate length	31
Substrate width	31
Thickness of the substrate	0.7
Thickness of the patch and ground	0.11
Partial ground length	10
First ground notch size	3.6*10
Second ground notch size	1*7.6

The SAR, VSWR, and return loss were analyzed for each model to assess its effectiveness. Return loss and VSWR are dependent on the reflection coefficient $\Gamma$. The reflection coefficient ($\Gamma$) indicated the reflected power from the antenna.

6 $$\begin{aligned} \Gamma = \frac{V_0^-}{V_0^+}=\frac{z_L-z_0}{z_L+z_0} \end{aligned}$$

where $V_0^-$ is the reflected wave, $V_0^+$ is the incident wave, and $z_0 and z_L$ are the transmission line impedance and the load impedance respectively.

The standing wave ratio is a numerical representation of impedance matching between the antenna and the transmission line. It measures the ratio of the amplitude of the maximum standing wave $v_{max}$ to the minimum standing wave $v_{min}$ as shown in Equation (7). If the VSWR is under 2, an antenna match is typically considered satisfactory.

7 $$\begin{aligned} VSWR=\frac{v_{max}}{v_{min}}=\frac{1+\Gamma }{1-\Gamma } \end{aligned}$$

The specific absorption rate is the most suitable metric used in assessing the impact of EM field exposure in the very near field of a Radio frequency (RF) source [38]. The following equation was used to determine the local SAR measured in W/kg at any location in the human tissue:

8 $$\begin{aligned} SAR=\frac{\sigma E^2}{2\rho } \end{aligned}$$

where E is the amplitude of the electrical field in human tissue expressed in volts per meter (V/m), $\sigma$ is the conductivity of the tissue (in Siemens per meter, S/m), and $\rho$ is the density of the tissue (measured in kilograms per cubic meter).

### Breast phantom design

A range of breast phantoms was created to evaluate the practicality of the proposed antenna for detecting breast cancer. The Cole-Cole and Debye models are commonly employed to characterize the dielectric properties of biological tissues[39, 40]. Both models contribute to understanding how biological tissues interact with electromagnetic fields. The IT’IS material parameter database [41] was used with our model to determine the dielectric characteristics of the breast skin, fat, and glandular tissues in the desired frequency range, as shown in Table 1. The tumor properties were assumed according to the literature [42]. Computer Simulation Technology (CST) as a well-known software was used for electromagnetic simulation and analysis (Table 2).

**Table 2:** Breast phantom tissue dielectric properties

Tissue	Permittivity	Electrical	Density	Heat capacity	Thermal conductivity
		conductivity S/m	Kg/m^3^	J/Kg/ ^∘^C	W/m/ ^∘^C
Skin	34.1	2.34	1109	3391	0.37
Breast Fat	4.46	0.35	911	2348	0.21
Glandular	50	3.46	1041	2960	0.33
Tumor	54.9	4	1058	–	–

**Fig. 2:**
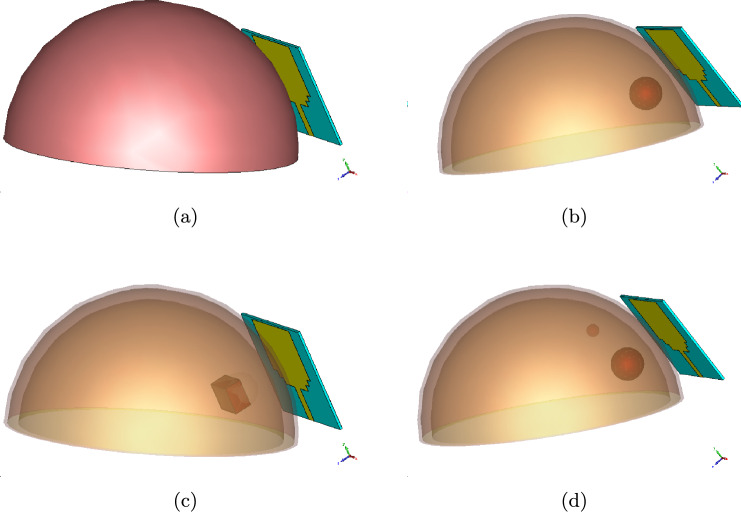
**a** Proposed antenna with the tumor-free breast phantom; **b** proposed antenna with a 5 mm-diameter tumor in the breast located at (0, $-$10,12); **c** proposed antenna with a 5 mm^3^ square tumor on a breast phantom and **d** proposed antenna with two tumors with diameters of 5 and 2 mm on the breast phantom

Figure 2 illustrates that the antenna design was initially tested on a breast model without tumors (Model A) and then on a breast model with a 5 mm radius tumor (Model B). Additionally, one more tumor with a 2 mm radius was placed in a different location in Model B to create Model C to evaluate the ability of the antenna to detect tumors in diverse areas. Finally, a square tumor was introduced to observe how it affected the antenna performance (Model D).

## Results

The return loss, SAR for radiation safety, voltage standing wave ratio to ensure antenna efficiency, and the impact of bending to determine flexibility, were examined and calculated for each model to measure the antenna performance.

**Fig. 3:**
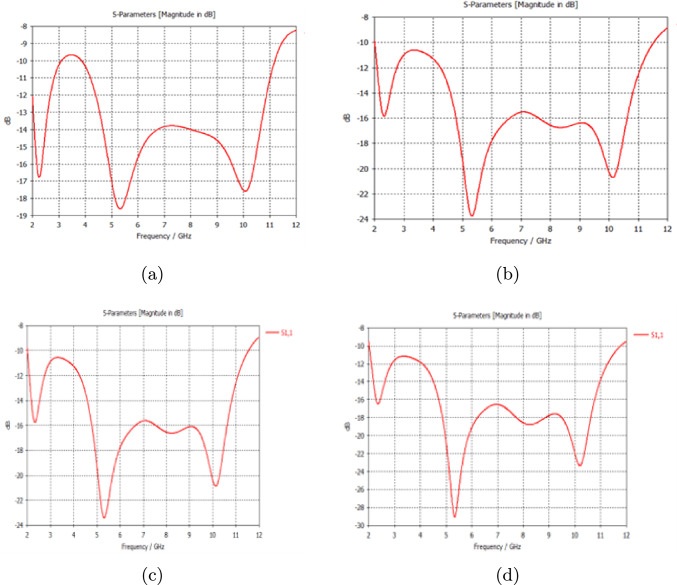
**a** S11 for Model A, **b** S11 for Model B, **c** S11 for Model C and **d** S11 for Model D

As shown in Fig. 3a, the model without a tumor gave a return loss of $-$18.5 dB. Figure 3b shows that there was an increase in the return loss from $-$18.5 dB to −24 dB due to the presence of a tumor. Figure 3c also shows a similar increase in S11, demonstrating that tumors with similar sizes and locations exhibited relatively identical increases in the return losses regardless of the form of the tumor. Despite the smaller size of the second tumor in Fig. 3d, which has two tumors of different sizes, there was a considerable increase in the S11 value compared to that of Model A, which indicated that the number of reflecting targets inside the breast affected the return loss directly.

**Fig. 4:**
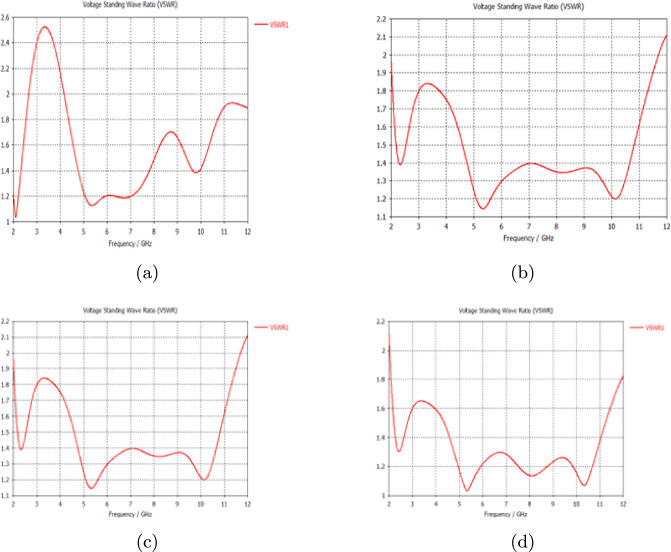
Simulated result of VSWR

Figure 4 shows that the VSWR values varied from 1.1 to 2 throughout the frequency 2–11.6 GHz range, indicating a satisfactory impedance match between the antenna and the transmission line.

**Fig. 5:**
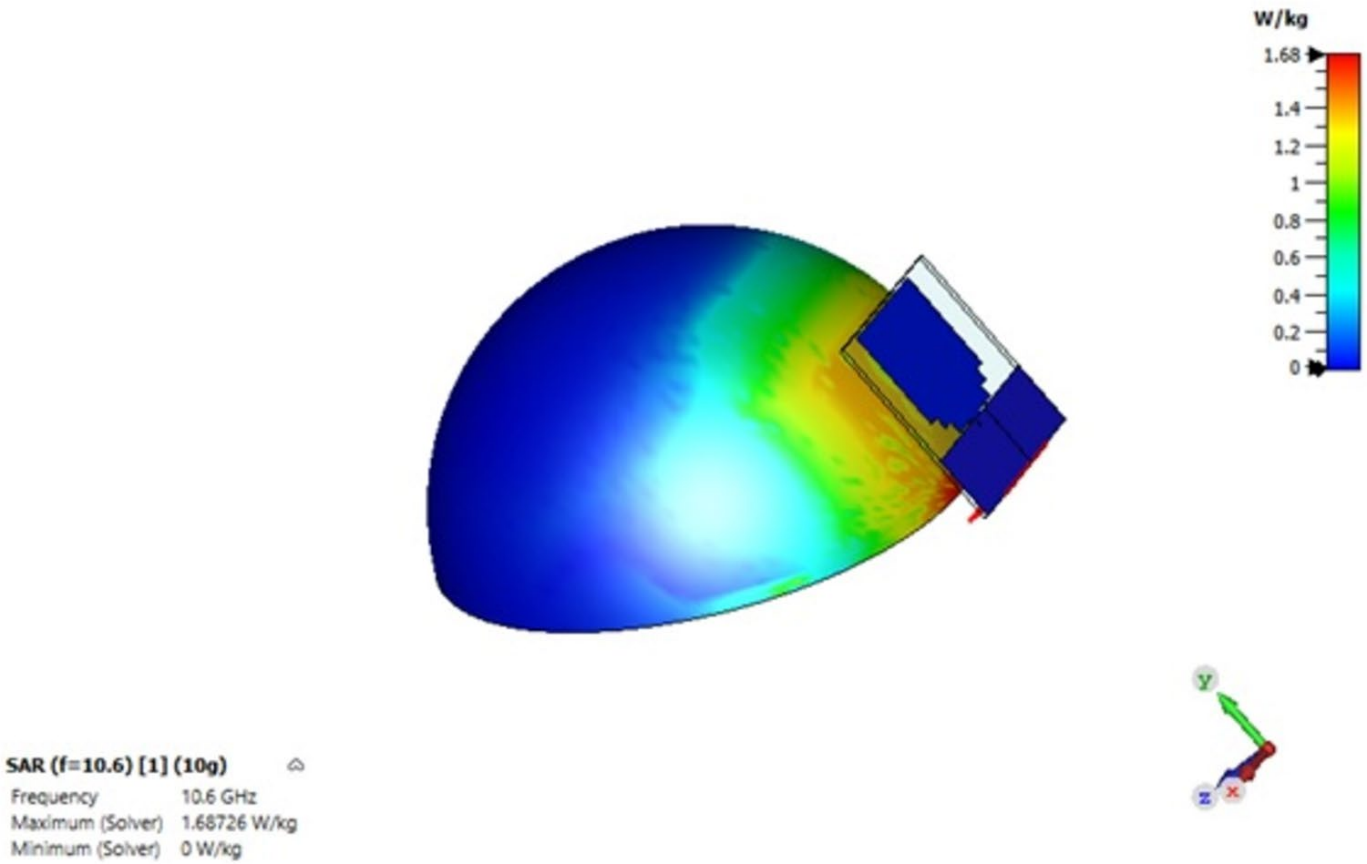
Obtained SAR value at 10.6 GHz

The specific absorption rate (SAR) values, which measure the impact of electromagnetic field exposure, remained within the allowed range for medical applications. The SAR varied between 1.6 and 2 W/g (10 g SAR) for the tested models, indicating that the antenna configuration was suitable for use safely in a breast cancer detection system. This is an important finding, as SAR values above the allowed range can cause harmful effects on human tissues which is a common problem in many previous research. Figure 5 shows the SAR value at a frequency of 10.6 GHz, which was calculated from Model B.

**Fig. 6:**
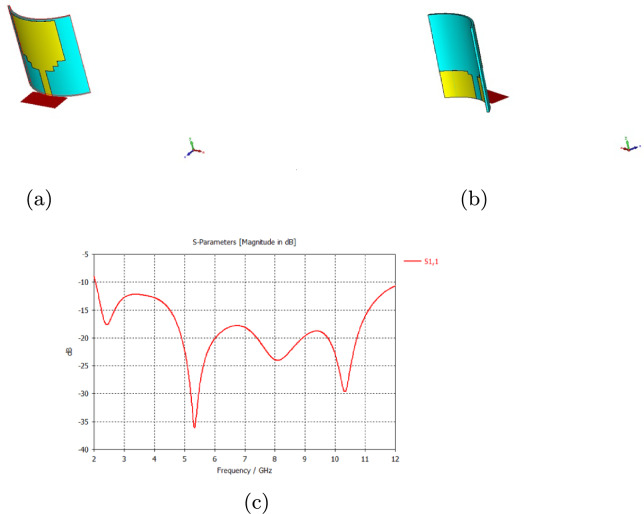
Bending effect on the antenna return loss

The suggested antenna was subjected to bending tests to determine its flexibility since the human body is not flat. Figure 6 shows the shifts in the resonance frequency arising from cylindrical bending but still working in the desired frequency range. This showed that the antenna maintained its performance even after bending, indicating its suitability for wearable applications. This is significant because the antenna must adapt to the body’s curvature for accurate detection.

## Discussion

In this study, a novel fully textile-based ultrawideband (UWB) microstrip patch antenna was designed, modeled, and simulated for early breast cancer detection. The antenna was tested with four numerical breast models with different tumor sizes, shapes, and locations to evaluate its performance. The results of the study showed that the antenna was effective in detecting breast tumors. The return loss, which indicates the reflected power from the antenna, was increased by the presence of a tumor. This increase was consistent regardless of the tumor shape, indicating that the antenna can detect tumors of different forms. The VSWR values remained within an acceptable range throughout the frequency range 2–11.6 GHz. The flexibility of the antenna was also tested by bending it. The antenna’s performance with respect to return loss, VSWR, SAR, and flexibility met the requirements for breast cancer detection.

**Table 3:** Comparison between the proposed work and some important research in the literature

Aspect	Proposed Work	Elsheikh et al.[35]	Lin et al.[32]	Mahmood et al.[27]	Hossain et al.[43]
Frequency range	2$-$11.6	1.8$-$2.4	1.198$-$4.055	7-28	2.42$-$3.2
(GHz)		and 4–10			and 4–15
Substrate/conductive	Denim/fabric	Cotton/roger-fabric	Polyester/copper	Denim/fabric	Felt/fabric
Antenna size (mm^2^)	31 $\times$ 31	24 $\times$ 41	40 $\times$ 45	60 $\times$ 50	80 $\times$ 61
Breast phantom	Multilayer	Single layer	Multilayer	Multilayer	Multilayer
SAR value (W/Kg)	Less than 2	Less than 2	Less than 2	Less than 2	0.121
Max S11 value (−10dB)	−36	−30	−40	−50	−50
Tumor size (mm)	2 and 5	10 and 20	None	None	None

An analysis presented in Table 3 reveals that although fabric materials can facilitate the attainment of a wide frequency range when employed as substrate and conductor as in [27]and [43], they invariably result in significantly larger antenna sizes. Notably, one of the primary challenges in Textile-based antenna design stems from the dielectric properties of these fabrics. It is essential to acknowledge that the antenna’s size plays a pivotal role in its suitability for integration into wearable imaging devices, particularly given the constraints imposed by the limited dimensions of the human breast. Smaller antenna dimensions facilitate their deployment within arrays. The primary achievement of this research lies in the successful development of a compact and lightweight antenna, capable of operating across a UWB frequency range utilizing fully textile-based materials. The designed antenna is small in size compared to the other antennas. On the other hand, as shown in Table 3, antennas with relatively small dimensions [32, 35] use metal-based conductive materials as patch and ground. Unfortunately, this design compromises flexibility and adaptability to conform to the body’s shape, making them less comfortable and potentially unsafe compared to fabric-based alternatives. Additionally, the antennas exhibit a narrower frequency range, which adversely impacts image resolution. It is worth noting that [28] succeeded in fabricating a smaller 20 x 20 antenna but suffered from a limited frequency range. Most previous research has used breast models composed of multiple layers that approximate the shape of the real breast. While these studies have proven successful in detecting spherical-shaped tumors, the current study goes beyond this by performing antenna tests to detect tumors of different shapes and sizes. According to that, this novel approach addresses a critical gap in existing literature. An important outcome of this research is the demonstrated capability to image tumors as small as 2 mm in diameter. Overall, the study demonstrated that the designed textile-based UWB microstrip patch antenna was effective in detecting breast tumors of various sizes, shapes, and locations. The use of a textile-based substrate provided comfort for the wearer, making it suitable for wearable applications. However, it is important to note that this study was based on numerical breast models, and further experimental validation is needed to confirm the antenna’s performance in real-world scenarios. The effects of other factors, such as different breast shapes, varying tissue properties, and the presence of other objects or clothing, must be investigated.

## Conclusion

This research was designed to develop a novel, inexpensive, comfortable, fully textile-based wearable UWB microstrip patch antenna capable of detecting breast cancer in its early stages. The antenna operated in the 2–11.6 GHz frequency range, which made it possible to attain good resolution at high frequencies above 5 GHz and good penetration at low frequencies. The proposed antenna comprised a jean substrate sandwiched between a patch layer and a ground plane made of a 100% polyamide-based fabric that had been metalized with copper, silver, and nickel.

The designed antenna was tested with four numerical breast models, and the tumor sizes, shapes, and locations were changed to test the ability of the antenna to detect the tumor. The use of tumors with different forms filled most of the gaps in the previous research, which considered the tumors to be spherical. The specific absorption rate (SAR), return loss (S11), and voltage standing wave ratio (VSWR) were calculated for each model to measure the antenna performance. The simulated SAR values remained between 1.6 and 2 W/g (10 g SAR), which were in the accepted range for medical applications. The VSWR remained within the acceptable range of 1.15–2. The return loss of the antenna varied from −36 to $-$18.5 dB for the four models and showed noticeable changes due to changes in the tumor size or location. The bending effect was examined to assess the antenna’s flexibility, and it showed good performance after bending. The provided antenna also did not require an immersion medium. As a result, the impacts of immersion-related imaging errors were eliminated, and the uncertainty in tumor localization was reduced. Fabrication and experimental measurement will be performed to validate the results of the designed antenna.
